# Addressing Parental Vaccine Concerns: Engagement, Balance, and Timing

**DOI:** 10.1371/journal.pbio.1002227

**Published:** 2015-08-07

**Authors:** Jason M. Glanz, Courtney R. Kraus, Matthew F. Daley

**Affiliations:** 1 Institute for Health Research, Kaiser Permanente Colorado, Denver, United States of America; 2 Colorado School of Public Health, University of Colorado Denver, Aurora, Colorado, United States of America

## Abstract

The recent United States measles epidemic has sparked another contentious national discussion about childhood vaccination. A growing number of parents are expressing concerns about the safety of vaccines, often fueled by misinformation from the internet, books, and other nonmedical sources. Many of these concerned parents are choosing to refuse or delay childhood vaccines, placing their children and surrounding communities at risk for serious diseases that are nearly 100% preventable with vaccination. Between 10% and 15% of parents are asking physicians to space out the timing of vaccines, which often poses an ethical dilemma for physicians. This trend reflects a tension between personal liberty and public health, as parents fight to control the decisions that affect the health of their children and public health officials strive to maintain high immunization rates to prevent outbreaks of vaccine-preventable diseases. Interventions to address this emerging public health issue are needed. We describe a framework by which web-based interventions can be used to help parents make evidence-based decisions about childhood vaccinations.

## Abstract 2

Vaccines save lives, yet an increasing number of parents in the US are hesitant to vaccinate their children. This Perspective proposes that intervention strategies to address this public health issue are needed.

**About the Author**

Jason Glanz, PhD**,** is an epidemiologist and senior research investigator at Kaiser Permanente Colorado, Institute for Health Research. He is also an assistant professor in the Department of Epidemiology, Colorado School of Public Health. Dr. Glanz’s research focuses on vaccine safety and vaccine hesitancy. Dr. Glanz is currently a principal investigator with the Vaccine Safety Datalink, a national Centers for Disease Control and Prevention (CDC)-funded project that examines the safety of vaccines. Dr. Glanz also leads research efforts to develop risk communication strategies to help reduce parental vaccination concerns.

## Introduction

In response to the recent US measles epidemic (that started in Disneyland of all places), vaccines have once again become the focus of a vigorous and contentious national discussion. The most recent controversy stems from evidence linking the outbreak to vacationing children who were intentionally unvaccinated, likely out of concerns that the risks of vaccination outweigh the benefits [1,2]. Media outlets sometimes refer to the parents of these children as “anti-vaxxers”—a pejorative term suggesting that such parents are antagonistic towards science. In public health, we prefer to use the term “vaccine hesitant,” which has evolved into an ill-defined concept covering any parent with concerns about the safety of childhood vaccination. However we characterize these parents, vaccine concerns are leading to behaviors that threaten the health of children and the communities they live in.

The issue of vaccine hesitancy can be examined from several different angles, many of which point to the delicate balance between personal liberty and public health. On one hand, in this age of shared decision making in health care, parents want control over the decisions that affect the health of their children. Ideally, parents would like to be armed with accurate scientific information so that they can objectively weigh the benefits and risks of vaccination and make informed decisions about immunizing their children. Through this process, some would argue, parents should have the right to refuse vaccines for their children.

On the other hand, it is essential to maintain high immunization rates to keep vaccine-preventable diseases at bay. In the case of measles, approximately 95% of the population needs to be immunized to prevent outbreaks. High vaccination rates, in turn, provide protection to vulnerable populations, such as those who cannot receive vaccinations for medical reasons, those whose immune systems do not respond to vaccines (vaccine failure), and children who are too young to be vaccinated. Compulsory vaccination laws help ensure that these vulnerable populations are protected. Mandatory school immunization laws in particular have been instrumental in helping to eradicate or control diseases that were once responsible for thousands of hospitalizations and deaths in the US each year [3,4].

In the past, when vaccine-preventable diseases were still prevalent in the US, it was abundantly clear to parents that the benefits of vaccination greatly outweighed the risks. Although pockets of vaccine resistance have existed since the beginning of vaccination, confidence in our national immunization program was high—physicians recommended vaccines, schools required them, and parents vaccinated their children [5]. Today, public trust in this model appears to be eroding to some extent. While vaccination remains the norm and immunization rates in the US are at an all-time high, national survey data show that greater than 20% of parents have concerns about the safety of childhood vaccination [6,7]. Twenty states now permit personal belief exemptions to school immunization requirements, which allow parents to opt out of vaccinations at school entry if they have a “philosophical” objection to them. Over the last two decades, a growing number of parents have been choosing to refuse or delay vaccines for their children. Between 10% and 15% of parents are requesting what are known as “alternative immunization schedules,” in which the spacing and timing of the vaccines are determined by the parent and not based on scientific evidence [8,9]. Research has shown that children on these alternative (or delayed) schedules are at greatly increased risk for pertussis, pneumococcus, and varicella infection [10–12].

Requests for alternative schedules pose an ethical dilemma for physicians. They have to simultaneously respect the parent’s right to choose and consider what is best for the health of the child and community. Persuading parents to immunize can be an uphill battle, as parents may come to check-up visits armed with information gathered from the internet, books, and other nonmedical sources that question the safety of vaccines. This prompts a negotiation between physicians and vaccine-hesitant parents, and physicians often have to invest substantial time during the 20-minute check-up visits trying to refute the misinformation. These visits may, in turn, end with the child leaving the doctor’s office underimmunized, having received only a subset of the recommended vaccines [13–17].

The increasing prevalence of parental vaccine concerns and the pressure it places on doctors are clearly pressing public health issues. Medical providers, policy makers, and researchers are all trying to figure out how to influence parental immunization attitudes and behaviors. It’s becoming apparent that we likely need better doctor–patient communication strategies, public health messaging campaigns, reliable web-based resources for parents, and stronger mandatory immunization laws. Recently, there have been vigorous discussions centered around strengthening state-level school immunization mandates. For example, in California, a bill (SB277) that eliminates personal belief exemptions to vaccination faced strident, vocal opposition as it progressed through the state legislature [18]. Although the bill was recently signed into law, political obstacles may be insurmountable in other states. For the other strategies to boost vaccine acceptance, we need interventions designed to test whether or not the strategies assist parents in the decision-making process for vaccination.

## Testing Strategies to Help Parents Make Evidence-Based Decisions about Vaccines

Numerous intervention studies have examined approaches to encourage vaccination. These include online decision aids, reminder/recall systems, patient and provider education, provider communication techniques, and financial incentives [16,19–24]. However, systematic reviews (including a review of published reviews) concluded that convincing evidence to support any intervention to specifically address vaccine hesitancy is lacking [25–27]. Recent studies show that strong provider recommendations and individually tailored approaches may be effective in increasing vaccine acceptance [28,29]. However, the results from these investigations are preliminary and need further study. In general, most studies in this area have either been observational or underpowered (i.e., small sample sizes) or have measured immunization attitudes and intentions rather than actual immunization behaviors as outcomes.

At Kaiser Permanente Colorado, we are one of several research groups that have started to develop and test interventions targeting parental vaccine hesitancy. We are currently conducting a randomized intervention trial to evaluate the effectiveness of a web-based resource that applies interactive social media technologies (blogs, discussion forums, and chat rooms) to engage parents, allay their concerns, and affect their immunization behavior. The development of this intervention was informed by a theoretical framework, existing evidence, and data collected from focus groups, surveys, in-depth interviews, and usability testing with a diverse group of parents [14,30]. Through this formative research, we have identified and have been applying the following themes in our intervention study: engagement, balance, and timing.

### Engagement

Increasingly, social media interventions are being used to promote health and behavior change across an array of health domains, including weight loss, smoking cessation, physical activity, and sexual health [31]. One theoretical basis for using social media is the multidirectional communication model, a social marketing framework that specifies a top-down, side-to-side, and bottom-up process for creating, presenting, and sharing information [32]. Top down refers to a traditional approach (web 1.0) in which web developers simply present information to users. Side to side and bottom up represent dynamic communication modes by which users can create and share content (web 2.0). The objective is to empower and engage users through open discussion and idea sharing.

The internet is rife with vaccine information that ranges from supportive to highly critical. Typically, vaccine advocates have employed the top-down approach to disseminate evidence-based information. These online resources are often sponsored by government agencies, public health departments, pharmaceutical companies, universities, and advocacy groups. Survey research has shown, however, that vaccine-hesitant parents in particular tend to be distrustful of such sponsors [33–35,6]. Moreover, the websites primarily present factual information that may seem dry and impersonal, and the extent to which they can engage parents is limited. While the Centers for Disease Control and Prevention (CDC) has developed a detailed website with useful information, parents in our studies have told us anecdotally that they find it difficult to navigate and wish that it had outlets to ask questions or interact with other users. It is therefore unclear if such top-down information-focused resources have a positive impact on parent decision making in regards to vaccines [26]. In fact, a recent study showed that presenting vaccine-hesitant parents with evidence-based information from government health agencies actually decreased their intentions to vaccinate [36].

Antivaccination advocates, in contrast, tend to use side-to-side and bottom-up approaches to present their messages on the internet. These groups are particularly adept at using social media to spread misinformation, often in the form of narratives and personal testimonials to appeal to parents’ emotions [37]. There are also numerous parenting blogs and discussion forums that promote conversations about vaccines among online communities of anonymous users. While the impact and reach of these forums is not known, they represent a potential source of misinformation that could be influencing parents’ vaccine decisions. In recent years, responding to vaccine concerns has felt like the old arcade game of “whack-a-mole”—a new concern arises, it gets refuted with science, and another new concern quickly pops up to take its place. It seems like antivaccine groups have been able to exacerbate this cycle with the internet and social media.

All of these factors led us to develop a hybrid intervention approach that combines evidence-based vaccine information with interactive outlets for parents to discuss vaccine-related topics with physicians, experts, and other parents. This type of web-based resource could not only empower parents by encouraging them to ask questions and voice their concerns but also allow website staff to quickly quash new vaccine-related rumors as they arise. To maintain a respectful environment, the website staff would also have to carefully fact-check the content and moderate the forums. The objective is to establish rapport with parents, build trust, and combat misinformation. Of course, creating such an environment poses many challenges, and we are hoping that our intervention will provide some useful data on its feasibility and effectiveness.

### Balance

A child’s medical provider is frequently the most trusted source of health information for parents [4]. Our research has shown that even parents who refuse or delay childhood vaccinations express a high level of trust in their doctors’ advice on nutrition, behavior, and development [14]. However, as expected, these hesitant parents also tend to be skeptical of their doctor’s advice on vaccines. They further report that their child’s doctor is much more likely to describe the “pros rather than the cons” of vaccination, and parents express a desire for more “balanced” information [38]. To some parents, balance likely implies receiving just as much information on the risks as on the benefits of vaccination. To a medical provider, balance should reflect the state of the scientific evidence, which warrants placing much more emphasis on the benefits than the risks. Yet, when physicians discuss vaccines with parents, it may seem as though they are withholding information on the risks.

It is not entirely clear how to bridge the gap between parents’ and providers’ perceptions of balance. Although there is a vast literature on how to communicate risk to patients [39–41], vaccines pose some unique challenges. On the surface, conveying the benefits and risks of vaccination seems like it would be straightforward—vaccines are highly effective at preventing serious diseases and severe side effects are rare. However, because of wide-scale vaccination, most vaccine-preventable diseases are also very rare in the US. Children are generally at low risk for contracting a vaccine-preventable disease, and quantifying individual risk is difficult because it depends on numerous factors. A child’s risk for disease is often determined by where the family lives, where they travel, who the child plays with, and the child’s immunity. This implies that physicians may have to engage in lengthy, nuanced conversations in order to paint a truly balanced picture for hesitant parents. Such conversations would have to simultaneously cover the individual- and community-level benefits of vaccination and the seriousness of vaccine-preventable diseases, as well as the risks of side effects.

Given the complexity of the topic, communication gaps are to be expected. This complexity argues for additional training to help physicians accurately communicate risk data, in addition to trusted educational resources to help parents digest vast amounts of scientific information. One possible communication approach is to simply state the risks of vaccination. Explicitly acknowledging the risks—which are real but rare—may ease the concerns of parents who believe that information is being hidden from them and facilitate trust [38]. Based on our formative research, for example, parents were adamant that our intervention website prominently display and explain the known side effects of vaccination. Whether or not such an approach is persuasive remains to be formally evaluated in our ongoing intervention trial.

### Timing

Determining when parents should receive vaccine information is important to an intervention’s success. Our research has shown that most vaccine-hesitant parents start to seriously think about vaccines while pregnant [14,42–44]. Other parents told us that the first time they received vaccine information was at the routine check-up visits, in the form of vaccine information statements (VIS) created by the CDC. Parents further stress that receiving this information so close to the actual vaccination of their child does not give them enough time to fully process the information. Lastly, vaccine-hesitant parents report that their vaccine decisions are constantly evolving over time. Some parents who refuse or delay vaccines describe themselves as being on “hyper alert,” worried that their decisions may be putting their child at risk for infection. These data suggest that parents’ immunization beliefs are not necessarily entrenched and that interventions should be implemented early during pregnancy and continue throughout the first several years of the infant’s life.

## Conclusion

Resistance to childhood vaccination is a concerning public health issue. However, in attempting to address vaccine resistance, it’s important to keep in mind that parents are just trying to do what is best for the health of their children. Many parents who worry about the risks of vaccination sift through an immense amount of material on vaccines, and physicians often find themselves having to contest the misinformation during short check-up visits at which there are other important topics to cover. It’s clear that we need better ways to inform and engage vaccine-hesitant parents. While interactive web-based technologies represent a promising approach, researchers should continue to experiment with strategies that focus on engaging parents with thoughtfully presented, evidenced-based information. Ultimately, successful interventions will be those that build trust, reduce concerns about unfounded risks of vaccines, and help parents understand that vaccinating on schedule is in the best interests of everyone, including their own children.

## References

[1] Zipprich J., Winter K., Hacker J., Xia D., Watt J., Harriman K. Measles outbreak—California, December 2014-February 2015. MMWR Morb Mortal Wkly Rep 2015 2 20; 64(6):153–4. http://www.cdc.gov/mmwr/preview/mmwrhtml/mm6406a5.htm?s_cid=mm6406a5_w. Accessed 2015 Apr 3. 25695321 PMC4584705

[2] Majumder M. S., Cohn E. L., Mekaru S. R., Huston J. E., & Brownstein J. S. Substandard vaccination compliance and the 2015 measles outbreak. JAMA Pediatrics 2015 5;169 (5):494–5. 10.1001/jamapediatrics.2015.0384 25774618 PMC4476536

[3] Omer S. B., Peterson D., Curran E. A., Hinman A., & Orenstein W. A. Legislative challenges to school immunization mandates, 2009–2012. JAMA 2014 2 12; 311(6):620–1. 10.1001/jama.2013.282869 24519303

[4] Omer S. B., Salmon D. A., Orenstein W. A., deHart M. P., & Halsey N. Vaccine refusal, mandatory immunization, and the risks of vaccine-preventable diseases. New England Journal of Medicine 2009 5 7; 360(19):1981–8. 10.1056/NEJMsa0806477 19420367

[5] Poland G. A., & Jacobson R. M. Understanding those who do not understand: a brief review of the anti-vaccine movement. Vaccine 2001 3 21; 19(17–19):2440–5. 11257375 10.1016/s0264-410x(00)00469-2

[6] Kennedy A., Basket M., & Sheedy K. Vaccine attitudes, concerns, and information sources reported by parents of young children: results from the 2009 HealthStyles survey. Pediatrics 2011 5; 127 Suppl 1:S92–9. 10.1542/peds.2010-1722N 21502253

[7] Kennedy A., LaVail K., Nowak G., Basket M., & Landry S. Confidence about vaccines in the United States: understanding parents’ perceptions. Health Affairs 2011 6; 30(6):1151–9. 10.1377/hlthaff.2011.0396 21653969

[8] Dempsey A. F., Schaffer S., Singer D., Butchart A., Davis M., Freed GL. Alternative vaccination schedule preferences among parents of young children. Pediatrics 2011 11; 128(5):848–56. 10.1542/peds.2011-0400 21969290

[9] Glanz J. M., Newcomer S. R., Narwaney K. J., Hambidge S. J., Daley M. F., Wagner N.M., et al A population-based cohort study of undervaccination in 8 managed care organizations across the United States. JAMA Pediatr. 2013 3 1; 167(3):274–81. 10.1001/jamapediatrics.2013.502 23338829

[10] Feikin D. R., Lezotte D. C., Hamman R. F., Salmon D. A., Chen R. T., Hoffman R.E. Individual and community risks of measles and pertussis associated with personal exemptions to immunization. JAMA. 2000 12 27; 284(24):3145–50. 11135778 10.1001/jama.284.24.3145

[11] Glanz J. M., McClure D. L., O’Leary S. T., Narwaney K. J., Magid D. J., Daley M.F., et al Parental decline of pneumococcal vaccination and risk of pneumococcal related disease in children. Vaccine 2011 1 29; 29(5):994–9. 10.1016/j.vaccine.2010.11.085 21145372 PMC3026079

[12] Glanz J. M., Narwaney K. J., Newcomer S. R., Daley M. F., Hambidge S. J., Rowhani-Rahbar A., et al Association between undervaccination with diphtheria, tetanus toxoids, and acellular pertussis (DTaP) vaccine and risk of pertussis infection in children 3 to 36 months of age. JAMA Pediatr. 2013 11; 167(11):1060–4. 10.1001/jamapediatrics.2013.2353 24019039

[13] Diekema D. S. Responding to parental refusals of immunization of children. Pediatrics 2005 5; 115(5):1428–31. 15867060 10.1542/peds.2005-0316

[14] Glanz J. M., Wagner N. M., Narwaney K. J., Shoup J. A., McClure D. L., McCormick E.V., et al A Mixed Methods Study of Parental Vaccine Decision Making and Parent–Provider Trust. Acad Pediatr. 2013 Sep-Oct; 13(5):481–8. 10.1016/j.acap.2013.05.030 24011751 PMC3767928

[15] Kempe A., O’Leary S. T., Kennedy A., Crane L. A., Allison M. A., Beaty B.L., et al Physician Response to Parental Requests to Spread Out the Recommended Vaccine Schedule. Pediatrics 2015 4; 135(4):666–77. 10.1542/peds.2014-3474 25733753 PMC6046639

[16] Opel D. J., Heritage J., Taylor J. A., Mangione-Smith R., Salas H. S., Devere V., et al The architecture of provider-parent vaccine discussions at health supervision visits. Pediatrics 2013 12; 132(6):1037–46. 10.1542/peds.2013-2037 24190677 PMC3838535

[17] Wilson K., & Keelan J. Social media and the empowering of opponents of medical technologies: the case of anti-vaccinationism. J Med Internet Res. 2013 5 28; 15(5):e103 10.2196/jmir.2409 23715762 PMC3668617

[18] California Lawmakers Pass Vaccine Bill Amid Emotional Debate. The New York Times. 9 June 2015. http://www.nytimes.com/aponline/2015/06/09/us/ap-us-xgr-vaccines-california.html?_r=0. Accessed 12 June 2015.

[19] Shourie S., Jackson C., Cheater F. M., Bekker H. L., Edlin R., Tubeuf S., et al A cluster randomised controlled trial of a web based decision aid to support parents’ decisions about their child's Measles Mumps and Rubella (MMR) vaccination. Vaccine 2013 12 5; 31(50): 6003–6010. 10.1016/j.vaccine.2013.10.025 24148574 PMC3898271

[20] Szilagyi P. G., Bordley C., Vann J. C., Chelminski A., Kraus R. M., Margolis P.A., et al Effect of patient reminder/recall interventions on immunization rates: a review. JAMA 2000 10 11; 284(14):1820–7. 11025835 10.1001/jama.284.14.1820

[21] Henrikson N. B., Opel D. J., Grothaus L., Nelson J., Scrol A., Dunn J., et al Physician Communication Training and Parental Vaccine Hesitancy: A Randomized Trial. Pediatrics 2015 7; 136(1):70–9. 10.1542/peds.2014-3199 26034240

[22] Opel D. J., Robinson J. D., Heritage J., Korfiatis C., Taylor J. A., Mangione-Smith R. Characterizing providers’ immunization communication practices during health supervision visits with vaccine-hesitant parents: A pilot study. Vaccine 2012 2 8; 30(7):1269–75. 10.1016/j.vaccine.2011.12.129 22230593

[23] Fairbrother G., Siegel M. J., Friedman S., Kory P. D., & Butts G. C. Impact of financial incentives on documented immunization rates in the inner city: results of a randomized controlled trial. Ambul Pediatr. 2001 Jul-Aug; 1(4):206–12. 11888402 10.1367/1539-4409(2001)001<0206:iofiod>2.0.co;2

[24] Lawrence G. L., MacIntyre C. R., Hull B. P., & McIntyre P. B. Effectiveness of the linkage of child care and maternity payments to childhood immunisation. Vaccine 2004 6 2; 22(17–18):2345–50. 15149795 10.1016/j.vaccine.2003.10.038

[25] Sadaf A., Richards J. L., Glanz J., Salmon D. A., & Omer S. B. A systematic review of interventions for reducing parental vaccine refusal and vaccine hesitancy. Vaccine 2013 9 13; 31(40):4293–304. 10.1016/j.vaccine.2013.07.013 23859839

[26] Dubé E., Gagnon D., & MacDonald N. E. Strategies intended to address vaccine hesitancy: Review of published reviews. Vaccine. 2015 4 18. pii: S0264-410X(15)00505-8. E-pub ahead of print. 10.1016/j.vaccine.2015.04.041 25896385

[27] Jarrett C., Wilson R., O’Leary M., Eckersberger E., & Larson H. J. Strategies for addressing vaccine hesitancy–a systematic review. Vaccine. 2015 4 18. pii: S0264-410X(15)00504-6. E-pub ahead of print. 10.1016/j.vaccine.2015.04.040 25896377

[28] Opel D. J., Mangione-Smith R., Robinson J. D., Heritage J., DeVere V., Salas H.S., et al The Influence of Provider Communication Behaviors on Parental Vaccine Acceptance and Visit Experience. Am J Public Health. 2015 3 19:e1–e7. 10.2105/AJPH.2014.302425 PMC4566548 25790386

[29] Gowda C., Schaffer S. E., Kopec K., Markel A., & Dempsey A. F. A pilot study on the effects of individually tailored education for MMR vaccine-hesitant parents on MMR vaccination intention. Hum Vaccin Immunother. 2013 2; 9(2):437–45. 23291937 10.4161/hv.22821 PMC3859769

[30] Shoup J. A., Wagner N. M., Kraus C. R., Narwaney K. J., Goddard K. S., Glanz J.M. Development of an Interactive Social Media Tool for Parents With Concerns About Vaccines. Health Educ Behav. 2015 6; 42(3):302–12. 10.1177/1090198114557129 25413375 PMC7651778

[31] Laranjo L., Arguel A., Neves A. L., Gallagher A. M., Kaplan R., Mortimer N., et al The influence of social networking sites on health behavior change: a systematic review and meta-analysis. J Am Med Inform Assoc. 2015 1; 22(1):243–56. 10.1136/amiajnl-2014-002841 25005606 PMC4433372

[32] Thackeray R., & Neiger B. L. A multidirectional communication model: Implications for social marketing practice. Health Promot Pract. 2009 4; 10(2):171–5. 10.1177/1524839908330729 19372278

[33] Downs J. S., de Bruin W. B., & Fischhoff B. Parents’ vaccination comprehension and decisions. Vaccine 2008 3 17; 26(12):1595–607. 10.1016/j.vaccine.2008.01.011 18295940

[34] Freed G. L., Clark S. J., Butchart A. T., Singer D. C., & Davis M. M. Sources and perceived credibility of vaccine-safety information for parents. Pediatrics 2011 5; 127 Suppl 1:S107–12. 10.1542/peds.2010-1722P 21502236

[35] Jones A. M., Omer S. B., Bednarczyk R. A., Halsey N. A., Moulton L. H., Salmon D.A. Parents’ source of vaccine information and impact on vaccine attitudes, beliefs, and nonmedical exemptions. Adv Prev Med. 2012; 2012:932741 10.1155/2012/932741 23082253 PMC3469070

[36] Nyhan B., Reifler J., Richey S., & Freed G. L. Effective messages in vaccine promotion: a randomized trial. Pediatrics 2014 4; 133(4):e835–42. 10.1542/peds.2013-2365 24590751

[37] Kata A. Anti-vaccine activists, Web 2.0, and the postmodern paradigm–An overview of tactics and tropes used online by the anti-vaccination movement. Vaccine 2012 5 28; 30(25):3778–89. 10.1016/j.vaccine.2011.11.112 22172504

[38] Benin A. L., Wisler-Scher D. J., Colson E., Shapiro E. D., & Holmboe E. S. Qualitative analysis of mothers' decision-making about vaccines for infants: the importance of trust. Pediatrics 2006 5; 117(5):1532–41. 16651306 10.1542/peds.2005-1728

[39] Ahmed H., Naik G., Willoughby H., & Edwards A. G. Communicating risk. BMJ 2012 6 18; 344:e3996 10.1136/bmj.e3996 22709962

[40] Zipkin D. A., Umscheid C. A., Keating N. L., Allen E., Aung K., Beyth R., et al Evidence-based risk communication: a systematic review. Ann Intern Med. 2014 8 19; 161(4):270–80. 10.7326/M14-0295 25133362

[41] Fischhoff B. Communicating Risks and Benefits: An Evidence Based User's Guide. Government Printing Office; 2012.

[42] Vannice K. S., Salmon D. A., Shui I., Omer S. B., Kissner J., Edwards K.M., et al Attitudes and beliefs of parents concerned about vaccines: impact of timing of immunization information. Pediatrics 2011 5; 127 Suppl 1:S120–6. 10.1542/peds.2010-1722R 21502250 PMC4536578

[43] Saitoh A., Nagata S., Saitoh A., Tsukahara Y., Vaida F., Sonobe T., et al Perinatal immunization education improves immunization rates and knowledge: A randomized controlled trial. Prev Med. 2013 6; 56(6):398–405. 10.1016/j.ypmed.2013.03.003 23524116 PMC6450531

[44] Wu A. C., Wisler-Sher D. J., Griswold K., Colson E., Shapiro E. D., Holmboe E. S., & Benin A. L. Postpartum mothers’ attitudes, knowledge, and trust regarding vaccination. Matern Child Health J. 2008 11; 12(6):766–73. 17987370 10.1007/s10995-007-0302-4 PMC3344281

